# Practical Implications of the Millennial Generation’s Consumer Behaviour in the Food Market

**DOI:** 10.3390/ijerph20032341

**Published:** 2023-01-29

**Authors:** Anetta Barska, Julia Wojciechowska-Solis, Joanna Wyrwa, Janina Jędrzejczak-Gas

**Affiliations:** 1 Faculty of Economics and Management, University of Zielona Góra, Podgórna 50, 65-246 Zielona Góra, Poland; 2 Faculty of Agrobioengineering, University of Life Sciences in Lublin, Akademicka 13, 20-950 Lublin, Poland

**Keywords:** food marketing, Generation Y, food consumption, consumer food satisfaction, innovation

## Abstract

Generational theory assumes that generational cohorts develop similar attitudes and beliefs. The Generation Y/Millennials group is currently one of the most important generations in the market as they have a presence in the labour market with a high income of their own, which creates higher demand for products, especially in the food market which is very sensitive to consumer decisions. The aim of this study was to show the consumer behaviour of Generation Y in the market for innovative food products and to propose marketing models created on the basis of research on a Polish sample of Millennials. The research was conducted in the period before the COVID-19 pandemic on a group of 544 selected respondents. Descriptive statistics of the SPSS program were used to process the results obtained. Among the most important of the results was identifying the decision-makers who are purchasing innovative products and the influence of third parties on their decision. In the first instance, consumers look for innovative food products in large retail chains (hypermarkets and supermarkets), however, they pay attention to both the quality of the products on offer and the price. Values such as freshness and taste also play a role in their purchasing decisions. Sources of information about innovative products in the Polish food market include culinary blogs recommending innovative products, and the opinions of dieticians and nutritionists.

## 1. Introduction

Innovations are an important part of the further advancement of societies in general as well as companies in particular. Individuals can benefit from the advantages of innovations, while companies can maintain or increase their market share and profitability. Especially in the food sector, scientific or technological innovation is often an important element that can determine a company’s entry to another level [1].

The importance of Generation Y for the food products market in Poland is determined by the fact that this cohort accounts for around a quarter of the country’s population, thus forming a market segment of great importance for the food products market. The size and specificity of the behaviour of Generation Y has, and will have in the future, a very large impact on the functioning of the key supply entities of this market—food production enterprises, farmers (who have to decide on their farm’s specific specialisation) and other economic entities present along the supply chain. This provides the rationale for undertaking research not only to identify the behaviour of Generation Y consumers in the food market, but also the actions taken by economic operators to shape an offer that meets the expectations of this generation. The evolution of consumer attitudes and behaviours, both in the market and other areas of social life, is significantly influenced by the economic and social development of economies and structural and technological changes that constantly occur in the environment. Consumers’ needs and ways of satisfying them change, as does how decisions are made, which is the result of progressive globalisation and convergence processes on the one hand, and disintegration and divergent processes on the other. In this process, the development of new technologies and ways of communicating also play an important role.

Generational marketing is the practice of referring to the unique needs and behaviours of an individual belonging to a particular generation group. The generation considered in the article is Generation Y, or Millennials, whose members were born in the last two decades of the twentieth century. The behaviour of this purchasing group has been influenced by a long period of economic prosperity, political changes, rapid advancement of modern technology, and the globalisation process [2]. The specifics of Generation Y—their values, attitudes, and behaviour—make it necessary to update marketing strategy in terms of value offered to young consumers [3]. This also applies to the food products market where thorough research is required, especially since consumers can express ethnocentric and conservative attitudes towards these products [4] and also changes and development trends observed in the food sector in Poland [5]. In the process of building relations with millennial consumers, marketing experts should consider their specific characteristics and behavioural determinants. Problems are addressed in reference to the new paradigm of consumption, which approaches consumption subjectively, meaning factoring in the characteristics of consumer behaviour and responsibility.

The aim of this article is to present possible marketing activities considering the characteristics and targeting of Millennials in the food products market. A literature review was conducted as part of the research proceedings. In addition, selected findings were presented from a survey carried out on a sample of 544 millennial consumers, developed using exploratory factor analysis, among other tools, in the pre-pandemic period.

The research results presented not only contribute to understanding the behaviour of millennial consumers, but also have practical potential for application to the food market, especially for companies operating in border markets or planning to enter them. The acquired knowledge could be used by food entities to develop effective marketing strategies in target markets and, therefore, to grow their business. Differences in consumer behaviour in the surveyed markets could provide inspiration for the food sector to increase innovations in the market. Government and non-governmental institutions, whose aim is to instil specific attitudes and behaviours in consumers and strengthen the food sector’s innovativeness in general, also need this information.

The research led to the conclusion that the marketing of food products to Millennial consumers should build on the achievements of various marketing concepts in line with the philosophy of holistic marketing. This approach is determined primarily by the fact that the market it concerns is heterogeneous, where consumers express different expectations of the values associated with the consumption of food products.

## 2. Literature Review

A generation is defined as a group of people born and living in the same period [6]. According to G. Aniszewska, members of a given generation learn behavioural patterns that affect their attitudes later in life [7]. Each generation has unique expectations, experiences, lifestyles, and values, as well as demographic characteristics that influence their buying behaviour [8]. What sets different generations apart is their tendency to focus on the values with which they were raised. Consequently, consumers of a specific generation have similar habits, attitudes, expectations, behaviour, and susceptibility to various arguments and incentives.

The millennial generation (also known as Millennials or Generation Y) considered in this study grew up in different economic and technological conditions from their predecessors, and these conditions have moulded their relationship to their surroundings. They were born into a technological, electronic, and wireless society with global boundaries becoming more transparent. Efficient multi-tasking helps them be successful. Howe and Strauss describe Generation Y as optimistic, cooperative team players and rule followers. They present rational minds, a positive attitude, and selfless team virtue [9]. This is a generation that has witnessed significant political changes and has been largely influenced by new technologies. Millennials, as a generational cohort, are exerting increasing pressure on the market environment and prompting the evolution of several sectors of the economy. The millennial generation is considered the largest and best educated [10]. Nielsen defines Millennials as the “We, More and Now Generation”, with eight shared characteristics:

1. Aspiration to a healthy lifestyle and better products;
2. Desire for control and personalisation;
3. Multi-tasking (engaging in multiple activities at once often involving mobile);
4. Value authentic;
5. On-the-go and connected everywhere at all times;
6. Opening to sharing their experiences online are open to sharing their experiences online;
7. Seeking added benefits and perks;
8. Like convenience and ease-of-use [11].

In the process of building relationships with consumers of a given generation, marketing experts should account for their specific characteristics and behavioural determinants. Generational marketing is the practice of referring to the unique needs and behaviours of an individual belonging to a particular generation group. The aim of this article is to present possible marketing activities, which consider the characteristics and targeting of Millennials in the food products market. Secondary and primary sources of information were used in the research procedure, with a literature review and a survey of 544 residents.

### 2.1. The Specifics of Food Product Marketing Review of the Literature

Maintaining a sustainable competitive advantage in the food market is becoming increasingly difficult and market orientation could provide a solution for both producers (manufacturers) and distributors of food products. Market orientation entails the pursuit of profit by maximising consumer satisfaction. In the food market, an important element of ensuring this satisfaction is the product, but more importantly, the values arising from its acquisition and consumption. According to D.R. Lehmann and R.S. Winnie, a product is the set of features, attributes, and benefits offered in order to satisfy needs and desires in the process of bringing about an exchange [12]. For the customer, value is the sum of benefits/values in the economic, technical, psychological, and social sense provided by a product that comes with tangible and intangible attributes, less the price paid for the product, accounting for the effort put in to explore the offer and make a choice. It should be borne in mind, however, that when analysing products and deciding on whether to buy them or not, the customer takes into account not only the benefits that he or she will obtain from the product itself but other values related to its purchase [13]. The concept of value is a complex one, depending on a number of variables. Consumers in the twenty-first century no longer just buy goods or services but, rather, they invest in the value they perceive as a result of the product or service’s utility and the cost of its acquisition. Traditional product utility measures based on utility assessment through economic criteria such as price and range are becoming increasingly less useful. Currently, product utility is a measure of pleasure; the satisfaction that people experience through the act of consumption [14].

The target market of the food sector is a wide range of recipients with diverse requirements and preferences, the definition and unification of which is the basis for product design. It should be remembered that consumers, when making buying decisions, are guided primarily by their own subjective assessment of product features, plus the meaning that they themselves ascribe to them now and the meaning these products will have for them in the future [15]. Consumers’ attitudes are influenced by product characteristics which are a source of symbolic, sensory, utilitarian, and functional values. Therefore, a successful launch of a food product on the market will depend on satisfying all of the consumer needs [16]. The springboard for developing a value strategy in the food sector is in its unique sensory values (taste, smell, colour, appearance), price, high quality, and brand image [17]. Quality is one of the most important attributes of all food products. Creating a proper and safe food product is associated not only with a lack of visible defects but also with the consumer’s satisfaction and fulfilment of his or her expectations at the time of purchase. Hedonistic values of food are playing an increasingly important role in this respect [18]. Consumers are aware of diseases and related consequences, and are increasingly paying more attention to the type, quality, and safety of products they are buying. Maciejewski [19] showed that a poor purchase risk, which accompanies consumers, if perceived, constitutes an important determinant of purchasing decision-making. This increased nutritional awareness among consumers contributes to the growth of interest in foods that are good for health and well-being. Consumers are aware that their diet, lifestyle, and life circumstances are among the most important factors affecting the health and quality of human life. Thus, when assessing food products, they pay attention to the content of specific nutrients, as well as the presence or absence of certain ingredients [20,21]. Equally important are attributes such as the size of packaging, convenience of use, and ease of preparation. On the rise also is the significance of social attributes in products—for example, being environmentally friendly, bearing certificates of traditional and regional food, or observance of ethical behaviour by producers [1,22]. Creating a safe and quality product has become a priority for food companies, who prefer operating in a fully stable market. As Kowalska and Paździor note, the success or failure of a food company has to do with two interrelated, aggregated premises: the efficiency of marketing activities and the complex of external market and post-market conditions [23].

**Figure 1:**
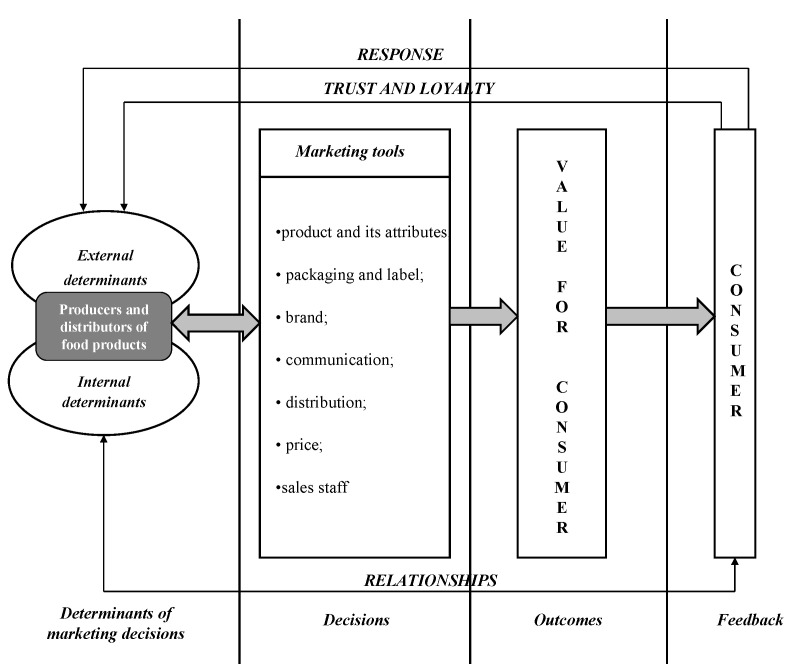
Building consumer satisfaction in the food market. Source: based on own research.

With this in mind, a proposition could be put forward to consider the marketing of food products as a total of all the decisions made by producers and distributors of food products resulting from specific external and internal circumstances [24]. The result would create value for the consumer, which would then enable the building of long-lasting relationships. Ideally, this approach should lead to increased trust and loyalty in customers and, ultimately, to more profits for the company (Figure 1).

Food marketing should therefore constitute a system of integrated activities aimed at creating and exchanging values in order to forge relationships between producers on one end and distributors, consumers, and other stakeholders on the other. These relationships should be analysed in the context of nutrition and health, symbols, economics, marketing, and social aspects [25].

### 2.2. Millennial Consumers in the Food Market

The millennial generation (also Millennials, or Generation Y) is a term referring to people born in the last two decades of the twentieth century. As such, they are a very large and rapidly growing market segment. In Poland, every fifth resident is a Millennial. Millennial consumers have a high purchasing power and therefore constitute the key segment for marketers. Generation Y is also called the digital generation; these “digital natives” appreciate all the technological faculties that facilitate the purchase of a product (applications, price comparison websites, simulations, culinary and nutritional advice, inspirations), and the ability to share opinions online on social media. Millennial consumer behaviours approach to purchasing or shopping goods differs to that compared with what was encountered by earlier generations. The millennial generation uses social media as a platform to communicate and shop. Millennials are more confident compared to earlier generations, more willing to be experimented on and to experiment themselves, and are conscious of trends [26]. They present rational minds, a positive attitude, and selfless team virtue.

A typical feature of Millennials concerns their high expectations towards the satisfaction of their needs [27]. They are self-absorbed and independent people, with a strong sense of individuality who willingly engage in social networks. They also display a great deal of narcissism and self-centredness [28]. A study by Solomon [29] showed a link between millennial socializing, entertainment, and consumption patterns; it follows that their interest in having fun and notably high spending ability is built on the notion that Millennials live for the moment and believe in enjoying themselves. Taken as a whole, Generation Y members are open, optimistic, goal-oriented, and highly driven to succeed. It is worth noticing that young consumers willingly accept innovations in the food market; every third respondent declared that he/she quickly buys innovative food products, but only after due consideration [30].

Williams and Page identified eight key values for Millennials: choice, personalisation, analysis, integrity, collaboration, speed, entertainment, and innovation [6]. The most crucial experiences of this generation are contact with new technology and excellent tech proficiency, greater mobility and openness, easier travelling and contact with other cultures, creation of virtual communities, a fast pace of life, and multi-tasking, but also impatience and the expectation of immediate acquisition, a change in approach to their own life—greater individuality, self-reliance, high self-esteem, and the pursuit of self-realisation [31]. Millennials tend to make quick purchasing decisions; they shop first for convenience and then for performance [32]. The pessimistic view of the millennial generation evaluates them as lazy, irresponsible, impatient, apathetic, selfish, disrespectful, and even lost, but the optimistic view labels them as open-minded, social, innovative, energetic, ambitious, confident, motivated, and smart. One common idea appears to be that they love to buy [33].

## 3. Materials and Methods

The aim of the research was to identify the values expected in an innovative food product by Generation Y consumers. The research results obtained provide the premise for the presentation of possible marketing activities which consider the specific characteristics and requirements of Generation Y and address these in the food product market.

The solution to any problem requires the establishment of a proper research procedure, which consists of a set of actions to be taken and performed in the right order to achieve the desired objective. The research was carried out in several stages, and the realisation of the main objectives of the research required action at two levels:

- Cognitive—analysing and critically interpreting the domestic and foreign acquis related to consumer behaviour;
- Empirical—conducting research aimed at identifying the attitudes and behaviour of Generation Y consumers.

Survey was chosen as the research method. The main research was preceded by exploratory research in order to create a standardised measurement tool. The survey questionnaire included questions of a closed alternative and multi-alternative nature. The scales used were mainly nominal and ordinal (Likert scale, rank scale). A pilot study also preceded the verification of the measurement tool. To assess the reliability of the measurement scales, Cronbach’s alpha test was used, which took the value from 0.748 to 0.888 as representing the correct reliability of the scales [34]. An exploratory factor analysis using the principal axis method was used to evaluate the empirical material posited. The factors were initially unknown and were extracted through analysis. They were grouped into sets of variables most strongly correlated with each other, and relationships between variables were detected without initial assumptions. The factor became a new variable that was not directly observable but was determined from the primary variables. Survey research counting with the participation of 544 Generation Y members living in the southwestern border areas of Poland. The size of the sample population was determined by that the total population in the study area, as well as the assumption of a confidence level of 0.95 and a 3% order of precision in the statistical inference of the fraction coefficient. The territorial scope of the research encompasses the border areas of Poland and was in line with the Cross-Border Friendship Database (CBFD), which also made it possible to use available secondary data to, inter alia, establish the size of the research sample, as well as other data depicting important phenomena (in terms of achieving the research objectives). The spatial scope of the research covered the following border voivodeships: Zachodniopomorskie, Lubuskie, Dolnośląskie, Opolskie, Śląskie, Małopolskie, and Podkarpackie. At the time of the study, these areas were inhabited by a total of 16,512,980 people (where Generation Y representatives accounted for 20.3%). The selection was made using the snowball method and thus was not random. In the study population, women made up 55% of the respondents and men 45%. When analysing the occupational activity of the respondents, it should be noted that two out of three respondents were economically active, often simultaneously studying, and without a permanent job. Every third respondent was economically inactive, such a state being the result of ongoing education or unemployment status. Every third respondent lived a rural area, while among city dwellers, representatives of small towns with up to 50,000 inhabitants prevailed. In terms of income, a significant number of respondents were without regular income from work and often dependent on their parents. This is due to the fact that many representatives of Generation Y are young people, still in education, or studying. Pilot studies were conducted on a group of 36 people.

## 4. Results

The research indicates the following characteristics of Millennials’ buying behaviour in the food market: A routine approach to shopping for food was predominant (65.2% of respondents):

- Shopping decisions are made individually (56.8%) and, when involving third parties, these are life partners (27.9%) or parents (12.5%);
- Preferred shopping places are hypermarkets and supermarkets (49.3% indicated these places as frequent shopping destinations) and discount stores (23.7%), while the prevailing criteria for their selection are the quality of inventory (weighted average of 3.73 out of 4), price level (3.60), product range (3.49), store location (3.40), and trust in the seller (3.31);
- The main criteria for selecting a food product are freshness (3.91), quality (3.75), taste values (3.73), and price (3.66);
- Nearly every third respondent determined his or her knowledge about innovations in the food market as either very good or good;
- Preferred informal sources of information about food products are own experiences and family or expert opinion, including nutritionists and specialist food blogs, whereas marketing sources include convenient locations and product-tasting stands.

**Figure 2:**
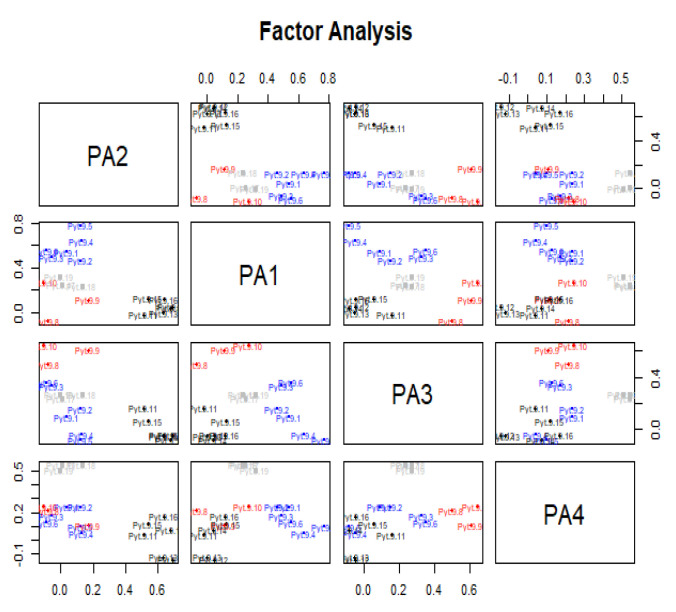
Reduced matrix of correlations. Source: based on own research.

Having reviewed the obtained data using exploratory factor analysis, the four main values expected of an innovative food product were distinguished. The main factors were initially unknown and extracted through analysis: the data were grouped into sets of variables most strongly correlated with each other without initial assumptions. The factor became a new variable that was not directly observable but determined from the primary variables. The number of factors and factor loadings were determined during the analysis. The resulting structure was interpreted only after the common factors were extracted [34] (Figure 2).

The review of the results began with an assessment of eigenvalues. For the four variables, the cumulative percentage of explained variance of the analysed variables was 83%, and no further variable explained more than 5% of the variance. A quartimax orthogonal rotation was used as the rotation method, which allowed the factor loadings for each variable to be determined and the loadings to be cleaned. Each factor was subject to interpretation due to primary variables which had high factor loadings. Primary variables without high loadings were removed from the analysis. The research procedure established a correlation of scores with factors (Table 1).

**Figure 3:**
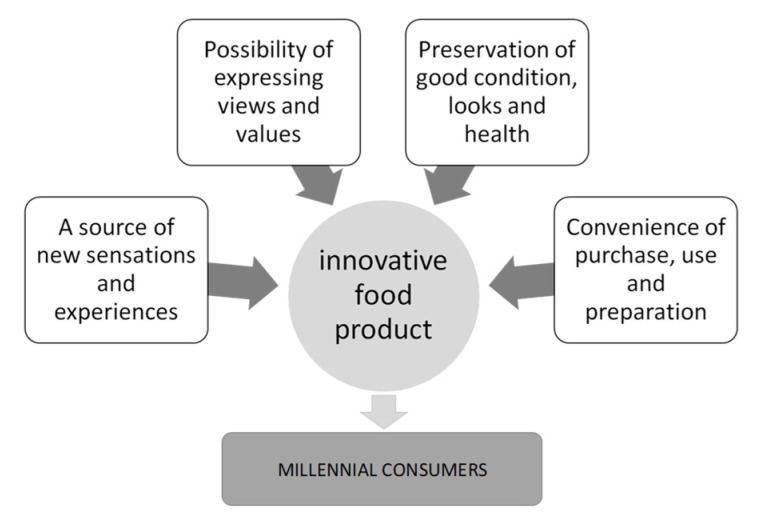
Main factors expressing the value of food products for the Polish consumer, determined based on exploratory factor analysis (the values of individual factors were 2.48, 1.67, 2.07, and 2.43, respectively). Identified factors explain approximately 83% of the total variance. Source: based on own research.

Based on the calculations performed in R, it can be seen that the first main factor (PA2), exhausting 46.4% of the total variability stock, is identified by variables related to the hedonic and cognitive values brought by the consumption of innovative foods. This factor relates to aspects of innovative foods, such as new sensations and experiences. The second main factor (PA1), describing 17.6% of the total variability stock, is primarily related to innovation as an opportunity to expose one’s views. The third factor (PA3) exhausts 11.5% of the stock of total variation and is identified by variables related to functional aspects of the food. It relates to aspects of the new food, such as health benefits or keeping fit and beautiful. The fourth factor (PA4) explains 7.5% of the total variability stock and is related to values such as the convenience of use and food product preparation (Figure 3).

A food product is the source of additional sensations and experiences that may result from its new taste and visual qualities and the high quality achieved by adding previously unused ingredients. Positive sensory impressions are essential attributes of food products, and this process must be accompanied by appropriate marketing information. Millennial consumers appreciate food products that positively impact the body (health and beauty). For example, the current fitness trend is an important driver for the food market, both in modifying existing recipes and developing entirely new ones. Many now desire easy-to-use products that will help them save time or be convenient to take. In addition, Generation Y consumers also appreciate the values arising from the social attributes of food products that allow them to express their views.

The marketing of food products to millennial consumers should build on the achievements of various marketing concepts in line with the philosophy of holistic marketing. This approach is determined primarily by the fact that the market it concerns is heterogeneous. According to the research, different expectations can be distinguished regarding the value of consumption of a food product, which requires the readjustment of an appropriate marketing policy (Figure 3). Food product marketing should combine elements of strategic marketing and, thus, focus on selected market segments, relationship marketing, and value marketing, using the assumptions of sensory marketing, lateral marketing, and experiential marketing. Marketing activities that target millennial consumers should concentrate primarily on the integrated concept of building values and relationships, which should prompt forging relationships with customers and making them more loyal. According to Furtak, relationships create value, and value builds relationships. Such relationships give a competitive edge, encourage purchase, and boost security and trust—factors that become the basis of the relationship at hand [35]. Value and relationships are mutually stimulating and interactive. Establishing permanent bonds is possible by using both attributes of food products, as well as emotional and sensory elements that are the sum of consumer experiences derived from contact with the company. Relationship and value marketing aim to build relationships with customers and shape their loyalty, which is a very important task in targeting Generation Y due to their weak attachment to producers and distributors [26]. It also promotes the creation of long-term relationships with the broadly understood surroundings, which in turn ensures profits for the company. In order to create value and relationships, selected elements of lateral, experience, retail, sustainable, and social marketing can be combined, which enables the development of an integrated, holistic marketing concept that makes it possible to stand out in the market. Sensory marketing applications allow the involvement of all sensory receptors of the consumer (eyes, ears, nose, tongue and palate, skin) in order to induce positive sensations leading to sensory satisfaction and subsequent purchase [36]. In the functioning of the food market, it may also be useful to implement the assumptions of lateral marketing [37], interpreted as a process that leads to the development of new, innovative products that consider the new needs of target customers and circumstances (location, time, situations, and applications). Meanwhile experiential marketing postulates looking at the market through the eyes of the consumer and providing them with experiences that will engage them and stay in their memory, thereby building a lasting relationship between the buyer and the company. It is worth emphasising at this point that experiential marketing—also referred to as engagement marketing—cannot, in practice, rely on sensory marketing only, but events must also be organised to accompanying the promotion of products (e.g., samplings). Positive sensory impressions are indispensable in the process of purchasing food products—a process that must go hand in hand with appropriate marketing information using both personal and impersonal information sources. It is worth noting that Generation Y consumers, despite the media’s strong influence, value family as a source of information. It means that they are strongly swayed by the circumstances of their native culture, which is a slightly different phenomenon in relation to other products [26]. Using the whole spectrum of communication tools to promote food products makes it possible to take advantage of the synergy effect. To improve efficiency and effectiveness, different created values can be combined, for example, convenience products, whose main determinants are the search for convenience and time savings, can be combined with the fitness trend. Consumers are happy to shop in places where they can purchase everything they need in one go, which is a challenge for the distribution activities of food producers.

**Figure 4:**
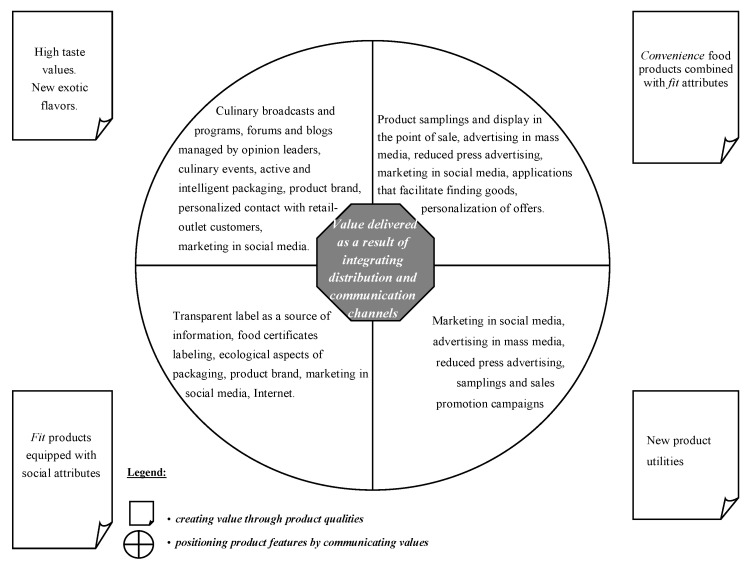
Examples of marketing activities targeting millennial consumers in the food market. Source: based on own research.

Food marketing which targets Gen Y consumers should comprise a set of integrated activities aimed at creating and implementing the exchange of values in order to create relationships between producers and distributors on one end and consumers and other stakeholders on the other (Figure 4). This approach may be particularly useful for local producers and distributors who are unable to compete on price with large retail chains.

## 5. Discussion

Across all sectors, it is essential for future-oriented companies to successfully develop and introduce new products to the market [38]. In order to develop new products, companies can fall back on scientific and technological innovations of various domains [39]. Consumers have adopted many of these technology-based innovations easily whereas other innovations have met with substantial resistance [40]. Within the food area, a similar picture emerges, with some recent technology-based innovations receiving high levels of consumer acceptance (e.g., nutraceuticals or fortified, enriched, or enhanced functional foods) [41,42,43].

What most strongly defines Generation Y is the emergence of modern technology and the digital revolution [44]. It is the first ever to be global—its reach extends to all who have access to the web [45,46]. For people of this generation, IT technologies are natural and necessary, an indispensable background, a necessary link to the surrounding world for real and/or virtual participation in it. For representatives of Generation Y, the Internet is not only a source of information but also a space for exchanging experiences and information [47,48]. They use modern technologies in every area of their lives and, thanks to Internet access, they are “citizens of the world”. The characteristics of Generation Y can also be examined through the prism of lifestyle, labour market choices, or purchasing behaviour. The specificity of these behaviours is conditioned, among other things, by the multichannel nature and unlimited availability of sales locations, the availability of sources of financing for purchases (interest-free retail, credit cards, short-term loans), and the vast amount of information reaching them through a variety of media [2,49]. The Generation Y consumer is characterised as being savvy and very well informed about market offerings through the use of modern technology to gather information [50,51]. Generation Y is described as a generation fascinated by food (“yum generation”, “foodies”, “yummers”). Shopping for food is a way to spend leisure time, preparing food is treated as a hobby, and eating food is a pleasure. Generation Y are pioneers in the use of unusual ingredients. Among this group of consumers, there is a trend towards healthy eating and they are in favour of “slow food”—spending more on food compared to previous generations by choosing quality food products that are definitely more expensive—and often eat out, while valuing home cooking experiments. They are interested in “bio”, “organic” and “natural” products [52,53,54]. Generation Y, also referred to as the digital generation, are “digital natives” who appreciate all technological facilities that facilitate product purchase (apps, price comparisons, simulations, culinary and nutritional advice, inspiration).

In Jaciow’s [3] study, friends and family and opinions of other buyers posted online influence the decision-making process. Generation Y buys products in the food market in the traditional way in stationary outlets, and online at shares and online shops. This is in line with the results of the authors of this study—a third party influences the decision to buy specific products by sharing their experience.

Referring to the research conducted by Barska and Wojciechowska-Solis [55,56] on the market for regional, traditional, and organic food products before the COVID-19 pandemic period, it is important to note the importance of stationary sales channels; only the pandemic period accelerated the acceptance of the online purchase channel. Similarly to innovative food products, the consumer often purchases required products in hypermarkets and supermarkets (this is supported by an extensive network of such shops offering food products in Poland) [55,57].

Variables such as the low price of products, their availability on shop shelves, and good quality and taste are undoubtedly assets of innovative foods in the market. Similar results were obtained by Mefleh et al. [58], studying the dairy market (between innovation and tradition). Variables indicative of high product quality are valued in any food market as evidenced by the results of the researchers mentioned above.

Charis et al. [59] as well as the authors of this paper, show that the current food consumer is an educated consumer. Their lockdown study showed that the consumer could distinguish between innovative products (i.e., knows what innovation is). The same results are presented by the Polish community of the surveyed millennial consumers who create demand for products on the market through their choices. As mentioned above, Generation Y is the age group that is currently in the labour market, which is manifested by higher earnings than other age groups. This is why this social group’s choices, attitudes, and preferences are so important for the food market, especially for companies wishing to enter the market with a new product.

The Polish Generation Y consumer takes into account the opinions of experts such as scientists, nutritionists, and nutritionists who blog. Experts are usually more open to innovative food technologies, as they appreciate the many benefits of using the innovation, such as improved food quality or simplified food production processes, more than the small uncertainties related to the potentially detrimental effects of the technology [60]. According to Siegrist [61], one key driver for a high consumer acceptance of food innovations is the consumer perception of the properties of those food innovations, which significantly influences the perceived risk as well as the perceived benefit of innovative food products. Over the past several years, numerous studies have identified several extrinsic factors that affect the perception of innovative food products [62,63,64,65,66]. These factors can be distinguished between features of the innovation to be adopted (e.g., innovativeness or naturalness), of the prospective consumer potentially adopting it (e.g., knowledge or moral concerns), and the social system in which the innovation is introduced (e.g., social trust) [39,67].

Worldwide institutions have carried out important actions for bridging education and capacity building towards innovation [66]. Organisations such as the Institute of Food Technologists (IFT) in the United States, the International Union of Food Science and Technology (IUFoST), the International Commission of Agriculture Engineering (CIGR), the ISEKI-Food Association, the Global Harmonization Initiative (GHI), and the International Dairy Federation (IDF) have been active and effective in congregating and disseminating scientific and technological knowledge by promoting the involvement of academy, private sector, and research institutes [68,69].

Progress in scientific and technological knowledge in different domains is enabling innovations for healthier and individualised food. Scientific advances in sciences allow for the definition of specific needs to provide products that attend to each consumer or group with common nutrition and functional requirements.

## 6. Conclusions

A food company wishing to operate and be successful in the market must skilfully evaluate its products, by considering its buyers’ prospects. Consideration of consumer behaviour in the generational approach is mainly due to the fact that the studied Generation Y was raised in different economic and technological conditions that have influenced their relationship with their surroundings. Millennials grew up in a different education model and under different authorities, which significantly impacted their expectations and behaviour, including consumption. Generation Y members are young, talented, and confident people raised in a world of new technologies. They cannot imagine living without computers, smartphones, and the Internet. Neither do they frequent traditional libraries, read newspapers in print, nor enjoy taking handwritten notes. These differences must not be ignored when planning marketing activities; the reason why the marketing of food products to millennial consumers should build on the achievements of holistic marketing by combining different concepts is to create the synergy effect. Attributes of the food product should be based on both tangible and intangible values since consumers also expect to be able to express their social preferences through their purchases. The broadly understood quality of food products is crucial for the consumer because it affects their health and is decisive when it comes to taste qualities. In marketing communication, not only digital media and community media should be used, but also expert channels and informal sources, as this will help reach millennial consumers more effectively.

The food sector could benefit from the insights provided by the results of this study to communicate and market their products accordingly to reduce mistrust and increase acceptance on the consumer side of food innovations.

Consumer-driven technology prioritises consumers’ demands, including sensory desires; therefore, their involvement is a key factor in the success of any innovation.

### Limitation

Due to the specific characteristics of innovative products, the sample presented does not reflect the country’s population. The main limitation of the study was that not all factors were considered. All variables included in this study were self-reported rather than observed. Due to the potential attitude–behaviour gap that is common in consumer research, the results should be treated with caution.

When conducting future research, all aspects (determinants) of consuming innovative products should be considered and particular attention paid to the importance of labels informing the consumer about the origins of the food, which, in this study, were treated marginally.

This study could serve as a starting (comparison) point for further research in the market for innovative food products. Additionally, the researchers intend to expand the research sample to include Z and alpha generations in the future.

An important limitation also stems from the sampling technique of the research sample, which was non-representative. This procedure was primarily determined by financial and organisational possibilities. Therefore, the results refer to the surveyed population and it cannot be assumed that they represent the opinions of the entire Generation Y population. In the future, increasing the territorial scope of the research and using random sampling would be valuable. In the course of the research, additional questions emerged that would require further exploration, especially given the permanent evolution of behaviour in times of civilisational change.

## Figures and Tables

**Table 1:** Measures of factor score adequacy.

	PA2	PA1	PA3	PA4
Correlation of scores with factors	0.90	0.88	0.81	0.73
Minimum correlation of possible factor scores	0.61	0.55	0.32	0.07

Source: based on own research.

## Data Availability

Not applicable.
